# Assessment of Spectra of the Atmospheric Infrared Ultraspectral Sounder on GF-5 and Validation of Water Vapor Retrieval

**DOI:** 10.3390/s21020325

**Published:** 2021-01-06

**Authors:** Xifeng Cao, Xiaoying Li, Shuanghui Liu, Xinyuan Zhang

**Affiliations:** 1 State Key Laboratory of Remote Sensing Science, Aerospace Information Research Institute, Chinese Academy of Sciences, Beijing 100094, China; caoxifeng18@mails.ucas.ac.cn (X.C.); liushuanghui19@mails.ucas.ac.cn (S.L.); zhangxinyuan20@mails.ucas.ac.cn (X.Z.); 2 University of Chinese Academy of Sciences, Beijing 100049, China

**Keywords:** AIUS, quality assessment, H_2_O profile, precision, vertical resolution, accuracy

## Abstract

Atmospheric Infrared Ultraspectral Sounder (AIUS) aboard the Chinese GaoFen-5 satellite was launched on 9 May 2018. It is the first hyperspectral occultation spectrometer in China. The spectral quality assessment of AIUS measurements at the full and representative spectral bands was presented by comparing the transmittance spectra of measurements with that of simulations. AIUS measurements agree well with simulations. Statistics show that more than 73% of the transmittance differences are within ±0.05 and more than 91% of the transmittance differences are within ±0.1. The spectral windows for O${}_{3}$, H${}_{2}$O, temperature, CO, CH${}_{4}$, and HCl were also analyzed. The comparison experiments indicate that AIUS data can provide reliable data for O${}_{3}$, H${}_{2}$O, temperature, CO, CH${}_{4}$, and HCl detection and dynamic monitoring. The H${}_{2}$O profiles were then retrieved from AIUS measurements, and the precision, resolution, and accuracy of the H${}_{2}$O profiles are discussed. The estimated precision is less than 1.3 ppmv (21%) below 57 km and about 0.9–2.4 ppmv (20–31%) at 60–90 km. The vertical resolution of H${}_{2}$O profiles is better than 5 km below 32 km and about 5–8 km at 35–85 km. Comparisons with MLS Level 2 products indicate that the mean H${}_{2}$O profiles of AIUS have a good agreement with those of MLS. The relative differences are mostly within ±10% at 16–75 km and about 10–15% at 16–20 km in 60${}^{\circ }$–80${}^{\circ }$ S. For 60${}^{\circ }$–65 ${}^{\circ }$ S in December, the relative differences are within ±5% between 22 km and 80 km. The H${}_{2}$O profiles retrieved from AIUS measurements are credible for scientific research.

## 1. Introduction

Water vapor is the main component of greenhouse gases. The change of water vapor in the stratosphere affects the radiation flux of long waves and short waves, as well as the temperature in the stratosphere and troposphere, which in turn plays a significant role in the balance of the global energy radiation budget. It has been proved that stratospheric water vapor contributes to global warming and may lead to more frequent extreme weather [1]. Water vapor serves as a dynamical tracer for monitoring the regional atmospheric circulation and the water circulation in real-time [2]. It occupies a large proportion of hydrogen in the middle atmosphere, which is mainly composed of H${}_{2}$O, H${}_{2}$, and CH${}_{4}$. Studying the exchange of hydrogen among different gases can provide valuable information for monitoring the physical condition of the atmosphere [3,4,5]. The distribution of water vapor in the polar regions is especially worth noticing, because water vapor enhancement in such a high altitude can promote Polar Mesospheric Cloud (PMC) formation by combining with the lowest temperature in the mesopause [3]. Therefore, understanding the spatial and temporal distribution of atmospheric water vapor and its long-term variation comprehensively is essential for our research on atmospheric dynamics, chemistry, and radiation.

Presently, many ground-based and satellite-borne sensors have been developed for H${}_{2}$O detection. Space-borne sensors observe H${}_{2}$O by nadir viewing, occultation detection, or limb sounding. Occultation and limb observation are less affected by the underlying surface and have high vertical resolution and sensitivity. Examples include Halogen Occultation Experiment (HALOE), Michelson Interferometer for Passive Atmospheric Sounding (MIPAS), Atmospheric Chemistry Experiment Fourier Transform Spectrometer (ACE-FTS), and Tropospheric Emission Spectrometer (TES) [6,7,8,9]. Atmospheric Infrared Ultraspectral Sounder (AIUS) aboard the Chinese GaoFen-5 satellite, launched on 9 May 2018, is the first hyperspectral occultation spectrometer in China. AIUS observes infrared occultation transmission for the retrieval of trace gases over the Antarctic, including water vapor (H${}_{2}$O). The main objective of this paper is to assess the AIUS spectral data and validate the H${}_{2}$O profiles retrieved from AIUS measurements. The overview of AIUS instrument is presented in Section 2. The Level 1 data quality assessment is presented in Section 3. The H${}_{2}$O retrieval and validation are described in Section 4. Finally, discussions and conclusions are given in Section 5 and Section 6.

## 2. AIUS Instrument

GF-5 is a sun-synchronous satellite orbiting at an altitude of 705 km, with an inclination of 98.2${}^{\circ }$. It has a mass of 2800 kg and a design life of 8 years. AIUS is a Michelson interferometer on the GaoFen-5 satellite with a spectral range of 750 to 4100 cm${}^{-1}$. The spectral resolution is 0.02 cm${}^{-1}$, which is similar to that of ACE-FTS in Canada. The measurements extend from the troposphere to mesosphere and span from 55${}^{\circ }$ S to 90${}^{\circ }$ S with a field of view of 1.25 mrad. AIUS can acquire information on many trace gases over the Antarctic [10]. Thus, it can provide sufficient data for weather forecasts and climate change research.

AIUS has a high spectral resolution and solar tracking accuracy [10]. The spectral resolution is mainly determined by the maximum Optical Path Difference (OPD) of the interferometer, and the spectral sampling interval is inversely related to maximum optical path difference [11]. Thus, a large OPD is needed to achieve hyperspectral resolution. A new design of interferometer that consists of double cube corner reflectors, an end mirror, a beam splitter, and a compensator applied magnifies the OPD eightfold. The specific sun-tracking camera and a two-dimensional pointing mechanism are used to provide fine pointing toward the radiometric center of the sun with a stability of 0.06 mrad. AIUS has two spectral bands: InSb and MCT. MCT and InSb detectors can obtain effective data in 750–2000 cm${}^{-1}$ and 1850–4100 cm${}^{-1}$, respectively. A more detailed description is presented in the document by Li et al. [12].

After data acquisition, both science data and auxiliary data are sent to the ground. The raw data (Level 0) are received at the ground station in binary format. Level 1 data processing is then performed to obtain transmittance.

## 3. Assessment of the AIUS Spectra

The spectral assessment of AIUS spectra was carried out by comparing the transmittance spectra of measurements with that of simulations. The Reference Forward Model (RFM) was used to simulate the transmittance. It has been widely used in ground-based, airborne, and space-borne observation [8,13,14,15,16,17,18]. The atmospheric profiles used in simulation were taken from the integrated atmospheric background dataset and were developed by our research group [19].

In this work, AIUS measurements from 60${}^{\circ }$–65${}^{\circ }$ S in June 2019 were applied to perform the comparison experiments. Because the data quality of the MCT band in 1850–2000 cm${}^{-1}$ is better than that of InSb, transmittances in 750–2000 cm${}^{-1}$ were taken from the MCT band, and transmittances in 2000–4100 cm${}^{-1}$ were taken from the InSb band. The mean differences and standard deviations of transmittance were calculated at different altitudes to quantify the differences between the AIUS data and simulated data.

The mean transmittance differences between the AIUS and simulated data are recognized as (**B**-**O**)${}_{mean}$. y${}_{AIUS}$ is the AIUS measurement, k is the number of AIUS measurements over the region of interest, and F(X${}_{i}$) is the simulated data. In the following context, the transmittance differences are simply expressed as differences.

(1) $${\left(B-O\right)}_{\mathrm{mean}}=\frac{1}{k}\sum_{i=1}^{k}\left[F\left(X_{i}\right)-y_{\mathrm{AIUS}\;}\right]$$

(2) $${\left(B-O\right)}_{\mathrm{std}}=\sqrt{\frac{1}{k}\sum_{i=1}^{k}{\left[F\left(X_{i}\right)-y_{\mathrm{AIUS}}-{\left(B-O\right)}_{\mathrm{mean}}\right]}^{2}}$$

### 3.1. The Comparison in the Whole Spectral Band

**Figure 1:**
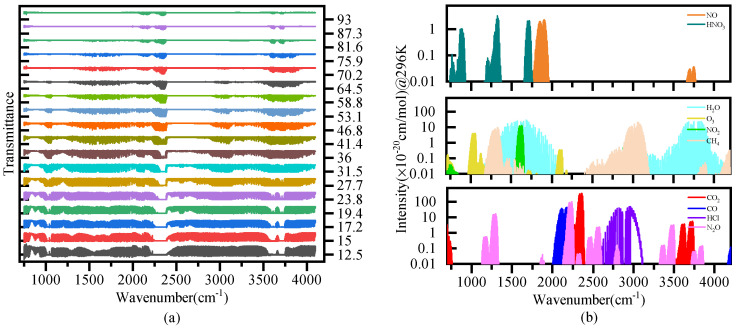
Atmospheric Infrared Ultraspectral Sounder (AIUS) measurement and the absorption line distribution in the 750–4100 cm${}^{-1}$.

After about seven months of the in-orbit test, AIUS have been continuously providing measurements [12]. Figure 1a shows AIUS spectra observed on 20 December 2019, where the horizontal axis is the wavenumber, and the vertical axis is the transmittance at different altitudes. In the 750–4100 cm${}^{-1}$ range, many components have characteristic absorption lines. Figure 1b shows the absorption line distribution of some trace gases (CO${}_{2}$, CO, HCl, N${}_{2}$O, H${}_{2}$O, O${}_{3}$, NO${}_{2}$, CH${}_{4}$, NO, and HNO${}_{3}$) in the 750–4100 cm${}^{-1}$ range. CO${}_{2}$, CO, and N${}_{2}$O have strong absorption lines, and the absorption intensity can reach up to 3.5 × 10^−18^ cm/mol.

**Figure 2:**
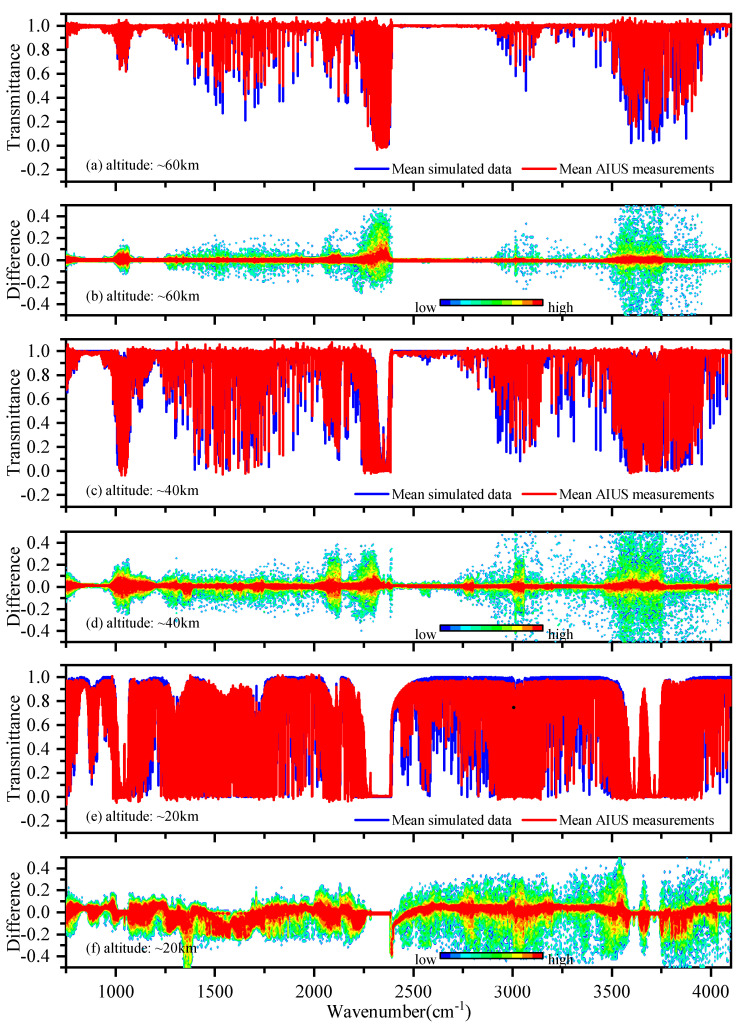
The comparison between mean AIUS measurements and the mean simulated data.

Figure 2 shows an overview of the transmittance differences between the AIUS observed data and simulated data in the spectral region of 750–4100 cm${}^{-1}$ for 60${}^{\circ }$–65${}^{\circ }$ S in June 2019. Thirty-two pairs of measurements and simulations were averaged at three altitudes (20 km, 40 km, and 60 km). In general, the differences at lower altitudes were larger than those at higher altitudes. This suggests that the differences were related to the increased pressure and pressure broadening at lower altitudes. Figure 2a,c,e shows the mean transmittance of observed data in the red line and that of the simulated data in the blue line. It is obvious to notice that the simulated spectra are similar to the measurements. Figure 2b,d,f shows the mean differences at different altitudes, where the color represents the point density. AIUS measurements and simulations agreed with each other. Most of the differences were within ±0.1. The lower differences were found in 800–1000 cm${}^{-1}$, 1070–1250 cm${}^{-1}$, and 2400–2900 cm${}^{-1}$ at 60 km, where the absorption was very weak. The larger differences can be seen in 980–1095 cm${}^{-1}$, 2250–2400 cm${}^{-1}$, 2800–3200 cm${}^{-1}$, and 3500–3750 cm${}^{-1}$. This might be due to the low signal-to-noise ratio (<200) in these spectral ranges and the uncertainties of the *a priori* profiles.

**Figure 3:**
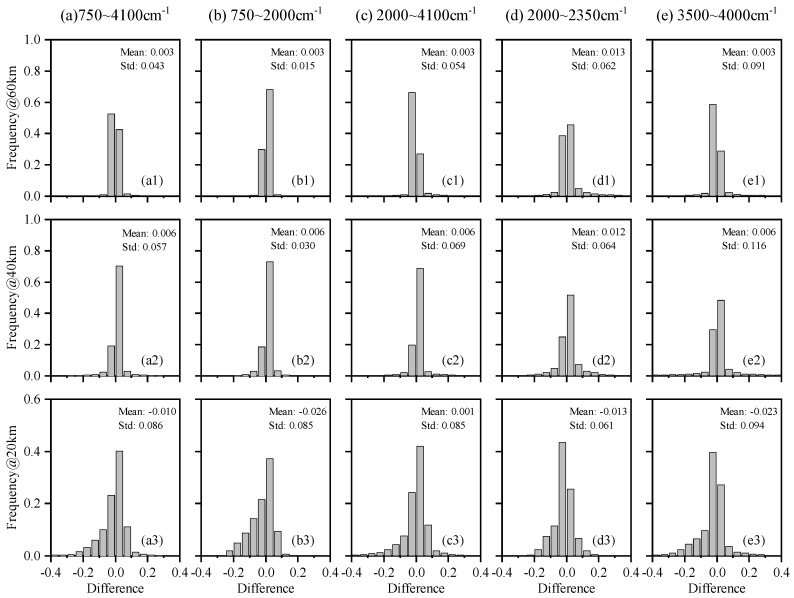
The frequency distribution histograms of the transmittance differences between AIUS measurements and simulations.

Figure 3 shows the frequency distribution histograms of the differences between AIUS measurements and simulations at three altitudes (60 km, 40 km, and 20 km) in five spectral bands. Figure 3a1–a3 shows the histograms for the full spectral band (750–4100 cm${}^{-1}$) of AIUS. There were 73% of the transmittance differences within ±0.05 at 20 km, 92% at 40 km, and 96% at 60 km. Figure 3b1–b3,c1–c3 are the histograms for the MCT and InSb bands. The histograms of the two bands were almost the same, which indicated that their detection ability was comparable. Figure 3d1–d3,e1–e3 are the histograms of two spectral bands (2000–2350 cm${}^{-1}$ and 3500–4000 cm${}^{-1}$, respectively) with large transmittance differences, as shown in Figure 2. There were more than 85% of the transmittance differences within ±0.15. Therefore, the AIUS measurements agreed well with the simulations.

**Figure 4:**
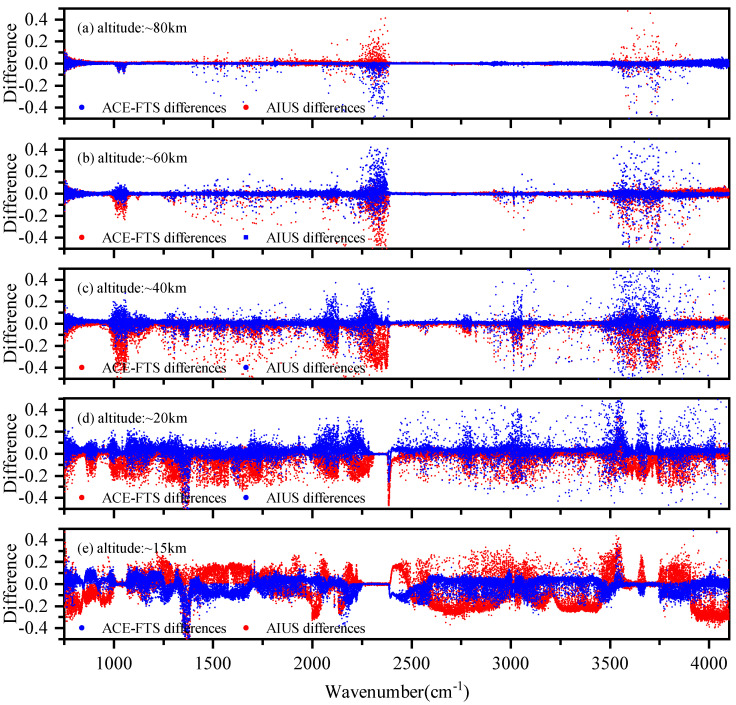
The transmittance differences between AIUS/ACE-FTS and simulated data.

As ACE-FTS has similar instrument characteristics, a comparison between the ACE-FTS measurements and simulations was also carried out. The AIUS and ACE-FTS measurement used in the experiment were acquired on 24 May 2019 (68${}^{\circ }$ S, 37${}^{\circ }$ E) and on 10 May 2012 (66${}^{\circ }$ S, 61${}^{\circ }$ E), respectively. As the acquisition time and location of the two measurements are not identical, a direct comparison of them is not allowed. The comparison is performed using simulations as the radiative transfer medium. The differences between the AIUS/ACE-FTS data and the simulated data are demonstrated in Figure 4. Figure 4a–d shows that the transmittance differences of ACE-FTS were similar to those of AIUS at 20–80 km, which indicates the transmittance of AIUS is mostly consistent with that of ACE-FTS and can provide reliable data for scientific research. Both sensors have a larger difference in 2250–2400 cm${}^{-1}$, 2800–3200 cm${}^{-1}$, and 3500–3750 cm${}^{-1}$. The differences between AIUS/ACE-FTS measurements and simulations were probably mainly due to uncertainties of the environment and the instrument during the observations that cannot be exactly simulated. Figure 4e shows that the differences of ACE-FTS are different from those of AIUS at 15 km, which indicates there is a problem with the AIUS transmittance spectra at 15 km. This might be due to the large noise level and uncertainties of the *a priori* profiles at lower altitudes.

### 3.2. Comparison in the H${}_{2}$O Spectral Window

The comparisons were also made between AIUS measurements and simulations in six spectral windows for O${}_{3}$, H${}_{2}$O, temperature, CO, CH${}_{4}$, and HCl. Here, we only present the comparison in the spectral window of H${}_{2}$O. The other comparisons are shown in Appendix A.

**Figure 5:**
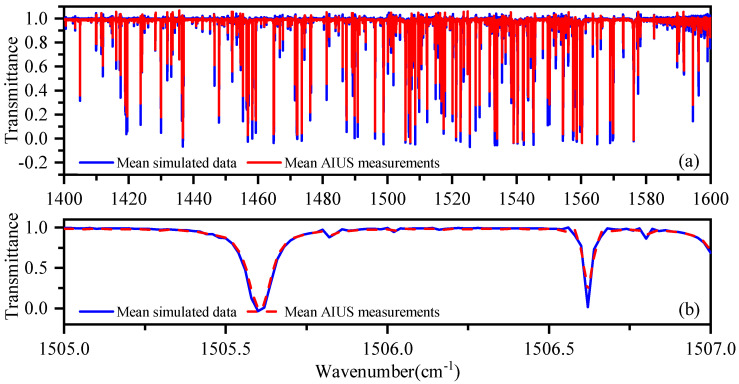
Mean transmittance of AIUS measurements and simulations in 1400–1600 cm${}^{-1}$ at 40 km.

Figure 5 presents the mean transmittance of AIUS measurements and simulations in 1400–1600 cm${}^{-1}$ at 40 km, where H${}_{2}$O and NO${}_{2}$ are the main absorbers. H${}_{2}$O can be detected and retrieved by this spectral window. The red dash line represents the measurement, and the blue solid line represents the simulation. Figure 5a indicates that the AIUS measurements have a good agreement with simulations. The transmittance of measurements were slightly larger than those of the simulated data around the absorption peaks. Figure 5b shows the spectra in a zoom-in window centered at 1506 cm${}^{-1}$. The absorption peaks can be seen clearly in it. The absorption peaks of measurements agreed well with those of simulations. Therefore, AIUS can provide reliable data for H${}_{2}$O detection and dynamic monitoring.

**Figure 6:**
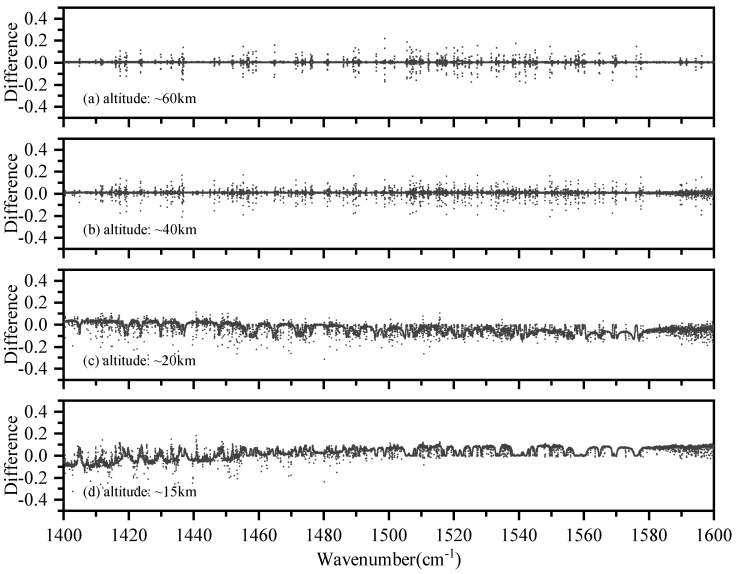
The transmittance differences between AIUS measurements and simulations in 1400–1600 cm${}^{-1}$.

Figure 6 shows the transmittance differences between the measurements and simulations in 1400–1600 cm${}^{-1}$ at 15 km, 20 km, 40 km, and 60 km, respectively. The differences vary in height and wavenumber. Figure 6a,b shows that the differences were within ±0.2 at 40 km and 60 km. Figure 6c illustrates that the differences were mostly in the range of −0.2 to 0.1 at 20 km. Figure 6d shows that the differences were in the range of −0.4 to 0.2 in 1400–1485 cm${}^{-1}$ and within ±0.13 in 1485–1600 cm${}^{-1}$ at 15 km. In general, the transmittance differences at higher altitudes were more discrete than at lower altitudes.

## 4. H${}_{2}$O Retrieval and Validation

### 4.1. Method of H${}_{2}$O Retrieval

AIUS has thousands of channels covering most of the infrared spectra. Before performing retrieval, an appropriate set of micro-windows has to be selected for H${}_{2}$O retrieval. The micro-windows should be sensitive to H${}_{2}$O and avoid the interference from other trace gases [20,21]. Sensitivity analysis and information entropy calculation are required [12]. The calculation of information entropy is given in Equation (3), where S${}_{i}$ is the covariance matrix of the ith iteration. A more detailed description can be found in Rodgers (1996) [22].

(3) $$H=\frac{1}{2}\left(\ln\left|S_{i-1}\right|-\ln\left|S_{i}\right|\right)=\frac{1}{2}\left(\ln\left|S_{i-1}{S_{i}}^{-1}\right|\right)$$

**Figure 7:**
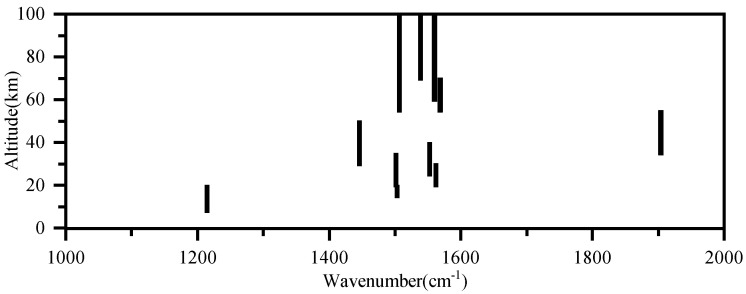
The selected micro-windows for H${}_{2}$O retrieval.

**Figure 8:**
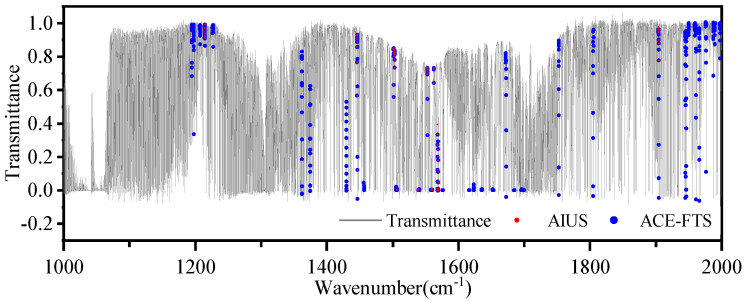
The comparison of micro-windows between AIUS and ACE-FTS.

Figure 7 shows the selected micro-windows for H${}_{2}$O retrieval at different altitudes. Eleven spectral micro-windows were selected in the 1000–2000 cm${}^{-1}$. The comparison of micro-windows between AIUS and ACE-FTS is shown in Figure 8. The solid line represents the transmittance spectra, the red dot represents the micro-window selected from AIUS, and the blue dot represents the micro-windows selected from ACE-FTS. The micro-window selected from AIUS were basically contained in those of ACE-FTS. Moreover, only 15% of the channels accounted for 75% of the information, which can improve the retrieval efficiency.

The optimal estimation method (OEM) was employed for H${}_{2}$O retrieval in this study [23,24]. The OEM retrieval framework has been used widely as it can solve the problem effectively of a nonlinear least squares minimization of a cost function [25]. The retrieval was initialized with the *a priori* profiles as the constraint, and the optimal atmospheric state was obtained through iteration. The H${}_{2}$O profiles were updated by Equation (4), where Y is the observation, X is the state of the atmosphere, F is the forward model, S${}_{a}$ is the covariance matrix of the *a priori* profiles, S${}_{e}$ is the covariance matrix of the observation error, and K is the matrix of “weighting functions” or “Jacobians”.

(4) $$X_{i+1}=X_{i}+{\left[\left(1+\gamma \right)S_{a}^{-1}+{K_{i}}^{T}{S_{e}}^{-1}K_{i}\right]}^{-1}\cdot \left\{{K_{i}}^{T}{S_{e}}^{-1}\left[Y-F\left(X\right)\right]-{S_{a}}^{-1}\left[X_{i}-X_{a}\right]\right\}$$

The AIUS H${}_{2}$O retrieval algorithm is presented in detail in the document by Li et al. [12].

### 4.2. H${}_{2}$O Profiles Retrieved from AIUS

In this study, 153 H${}_{2}$O profiles were retrieved from AIUS measurements obtained in 2019. The measurements were divided into four latitude zones of 60${}^{\circ }$–65${}^{\circ }$ S, 65${}^{\circ }$–70${}^{\circ }$ S, 70${}^{\circ }$–75${}^{\circ }$ S, and 75${}^{\circ }$–80${}^{\circ }$ S. Information about the AIUS measurements used in this study is listed in Table 1.

**Figure 9:**
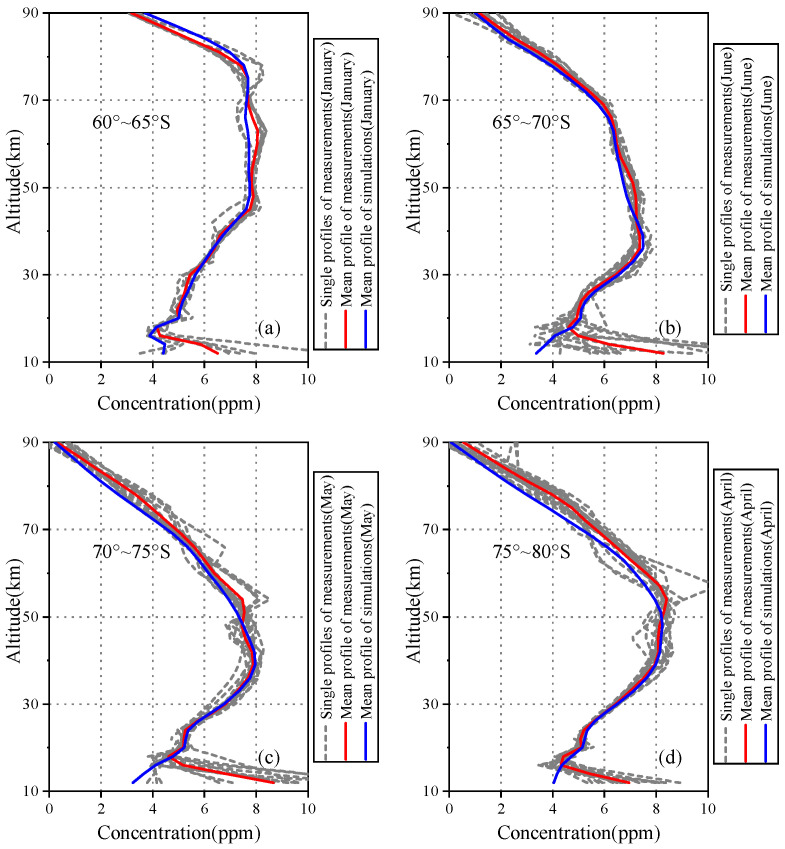
H${}_{2}$O profiles retrieved from AIUS measurements.

The H${}_{2}$O retrievals of representative months (January, June, May, and April) in four latitude regions are presented in Figure 9. The dashed line represents single profile retrieved from AIUS measurements, and the red line represents mean profiles retrieved from AIUS measurements. In general, the vertical distributions of the H${}_{2}$O mean profiles were different in different latitude zones and months, especially at the lower stratosphere and upper mesosphere. For 70${}^{\circ }$–75${}^{\circ }$ S and 75${}^{\circ }$–80${}^{\circ }$ S, the single profile distributions were more discrete than that in 60${}^{\circ }$–65${}^{\circ }$ S and 65${}^{\circ }$–70${}^{\circ }$ S, which indicated that the variance of the H${}_{2}$O profiles can be larger.

At the same time, the comparison of the H${}_{2}$O profiles retrieved from the AIUS measurements and simulations was carried out to reveal the impact of the transmittance differences between AIUS measurements and simulations. The blue line represents the mean profile of simulations shown in Figure 9. Figure 9a–d illustrates that the mean profile of AIUS is similar to that of simulations at 16–90 km. However, the mean H${}_{2}$O profiles of AIUS measurements deviate from that of simulations below 16 km. The biggest difference of H${}_{2}$O profiles was found at 12 km for 70–75° S in May, approximate 62%. The comparison of the AIUS spectra and simulation spectra illustrated that the transmittance differences were larger between the AIUS measurements and simulations at 15 km as shown in Figure 4e. Thus, the larger transmittance differences around 15 km can lead to greater differences of H${}_{2}$O profiles. The great uncertainties of measurement spectra and retrieval profiles at lower altitude might be introduced by the large noise level.

### 4.3. Validation of H${}_{2}$O Retrievals

The validation was carried out between the monthly mean profiles of AIUS and the equivalent Microwave Limb Sounder (MLS) Level 2 products. For MLS v4.2 H${}_{2}$O profiles, the effective vertical resolution is less than 3 km in 316–1 hPa and about 3.3–10.3 km in 1–0.002 hPa [26]. The precision is less than 35% in 178–0.046 hPa. The accuracy is within ±10% in 100–0.021 hPa, 10–25% in 316–100 hPa, and about 10–34% in 0.021–0.002 hPa [27]. The matching MLS and AIUS data was selected within 3${}^{\circ }$ latitude and 30${}^{\circ }$ longitude.

#### 4.3.1. Precision and Resolution

**Figure 10:**
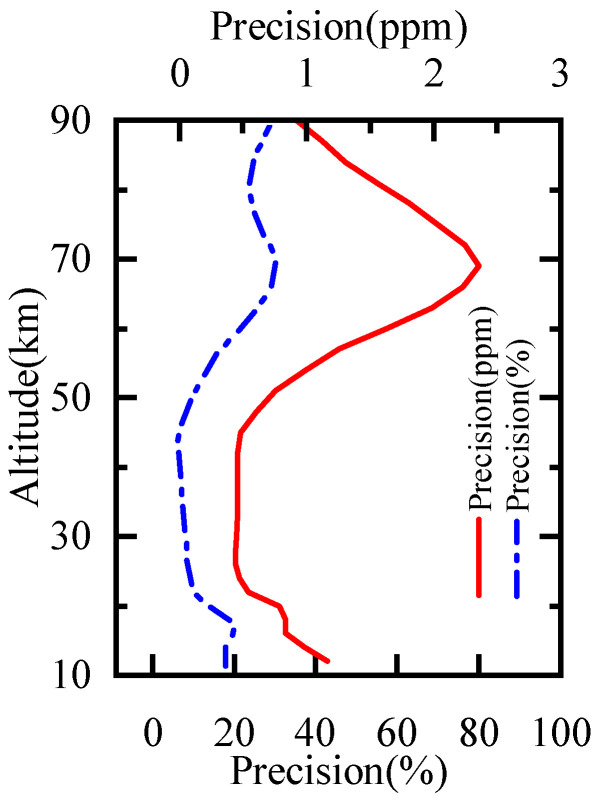
The precision of the H${}_{2}$O retrieval for 60${}^{\circ }$–65${}^{\circ }$ S in January.

The retrieval precision was calculated from the diagonal elements of the solution covariance matrix as Equation (5) [28,29]. The precision of H${}_{2}$O profiles for 60${}^{\circ }$–65${}^{\circ }$ S in January is shown in Figure 10. The red solid line represents the precision and the blue dash line represents the precision in percentage. The precision was less than 1.3 ppmv (21%) below 57km and about 0.9–2.4 ppmv (20–31%) at 60–90 km. It illustrated the precision of H${}_{2}$O profiles is comparable to that of MLS.

(5) $$S_{x}=\left(S_{a}^{-1}+K^{T}S_{e}^{-1}K\right)$$

**Figure 11:**
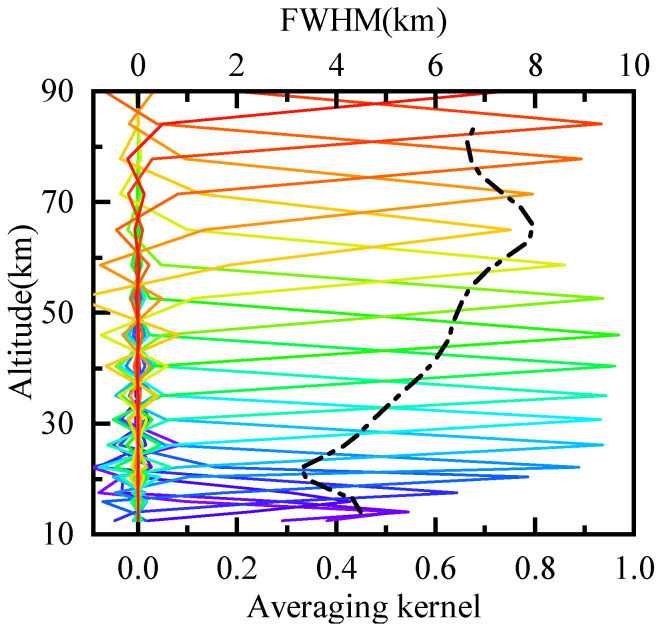
The vertical resolution of the H${}_{2}$O retrieval for 60${}^{\circ }$–65${}^{\circ }$ S in January.

The vertical resolution of the H${}_{2}$O retrieval was obtained from the full width at half maximum (FWHM) of the averaging kernels [29]. Figure 11 shows the averaging kernels and vertical resolution of H${}_{2}$O retrieval for 60${}^{\circ }$–65${}^{\circ }$ S in January. The solid line is the averaging kernel at 12–85 km with different color representing different altitudes. The dashed line represents the vertical resolution. The vertical resolution of H${}_{2}$O profiles was better than 5 km below 32 km, and about 5–8 km between 35 and 85 km.

(6) $$A=\frac{\partial \hat{x}}{\partial x}=\frac{\partial \hat{x}}{\partial y}\frac{\partial y}{\partial x}=G_{y}K={\left(K^{T}S_{e}^{-1}K+S_{a}^{-1}\right)}^{-1}K^{T}S_{e}^{-1}K$$

#### 4.3.2. Comparison with MLS H${}_{2}$O Product

For the comparison experiment, vertical resample with the same altitude grids was applied to both AIUS retrievals and MLS Level 2 products, from 12 km to 90 km, with 2 km steps below 30 km and 3 km steps over 30 km [12]. Horizontal interpolation was neglected because of a lack of high horizontal resolution correlative data [28].

(1) 60${}^{\circ }$–65${}^{\circ }$ S

**Figure 12:**
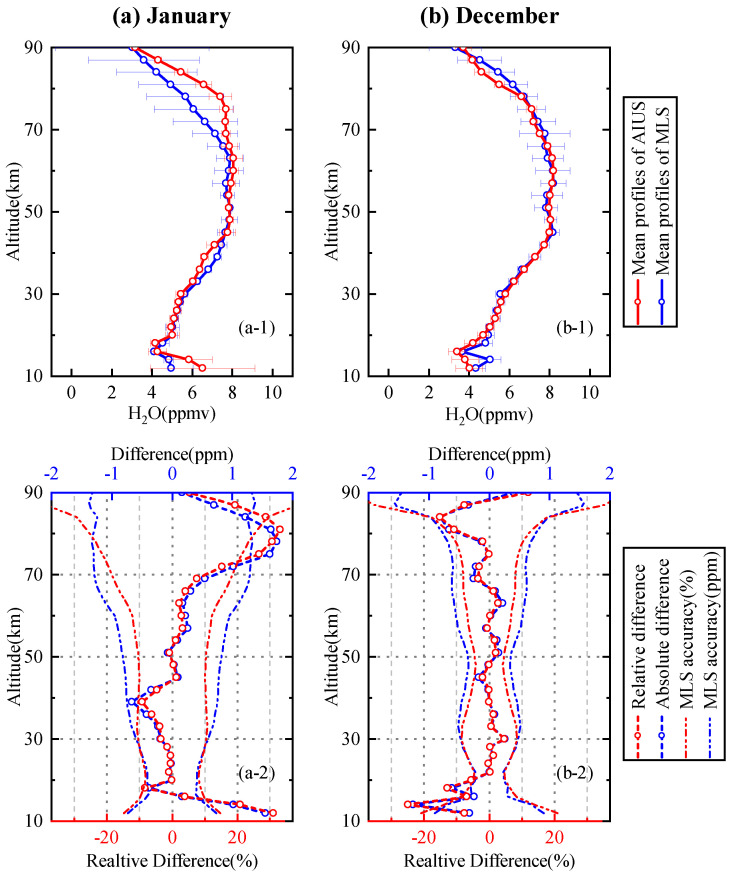
A comparison of H${}_{2}$O profiles between AIUS and MLS for 60${}^{\circ }$–65${}^{\circ }$ S in January and December: (**a-1**) mean profile and standard deviation in January of AIUS and MLS; (**a-2**) relative difference and absolute difference of mean H${}_{2}$O profiles between AIUS and MLS with MLS accuracy; Subfigure (**b**) is same as subfigure (**a**) except for December.

The comparison of H${}_{2}$O profiles for 60${}^{\circ }$–65${}^{\circ }$ S in January and December is presented in Figure 12. Figure 12a-1 shows the mean profile of AIUS and MLS in January. The mean profile of AIUS was consistent with that of MLS in 16–72 km. The standard deviations of the AIUS profiles were within 0.6 ppm in 16–90 km. The standard deviations of AIUS profiles were mostly less than those of MLS. The relative and absolute differences of the mean profile between AIUS and MLS in January are presented in Figure 12a-2. The relative differences were within ±10% (about 0–0.67 ppm) between 16 km and 70 km, and 4–33% at 10–16 km and 70–90 km. The relative differences stayed within the acceptable range of MLS differences at 16–72 km and 84–90 km. Figure 12b-1 illustrates that the mean profile of AIUS had a good agreement with that of MLS in December. The standard deviations of AIUS profiles were within 0.4 ppm between 16 km and 90 km. Figure 12b-2 shows that the relative differences were mostly within ±5% (about 0–0.3 ppm) at 20–80 km, and 5–15% at 16–20 km and 80–90 km. The relative differences basically stayed within the acceptable range of MLS differences at 16–90 km.

(2) 65${}^{\circ }$–70${}^{\circ }$ S

**Figure 13:**
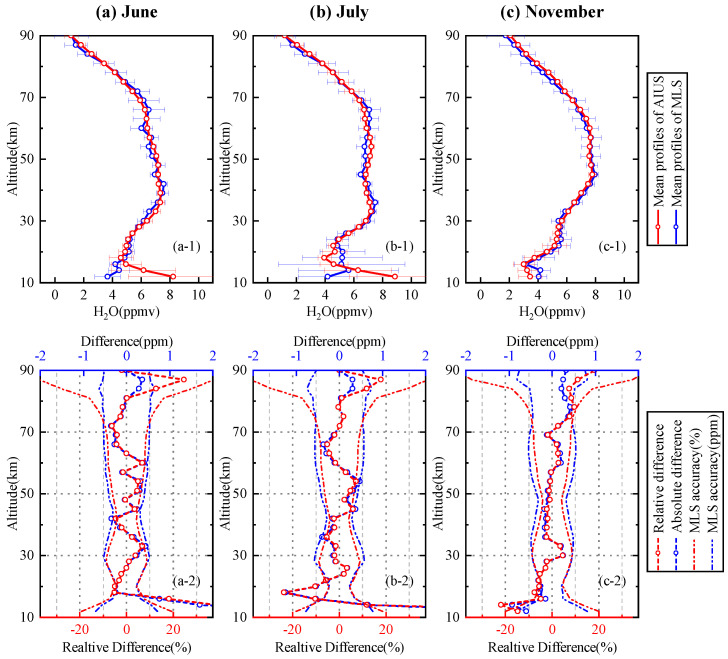
Same as Figure 12 except for 65${}^{\circ }$–70${}^{\circ }$ S in June, July, and November.

The comparison of H${}_{2}$O profiles for 65${}^{\circ }$–70${}^{\circ }$ S in June, July, and November are presented in Figure 13. Figure 13a-1 shows the mean profile of AIUS and MLS in June. The mean profile of AIUS agreed well with that of MLS. The standard deviations of AIUS profiles were within 0.5 ppm at 20–90 km. The relative and absolute differences of the mean profile in June are presented in Figure 13a-2. The relative differences were basically within ±7% (about 0–0.4 ppm) between 18 km and 81 km. Figure 13b-1 shows the mean profile of AIUS and MLS in July. The standard deviations of the AIUS profile were within 0.6 ppm at 18–90 km. Figure 13b-2 shows that the relative differences were basically within ±10% between 14 km and 84 km. Figure 13c-1 shows the mean profile of AIUS and MLS in November. Figure 13c-2 shows that the relative differences were less than 6% at 16–72 km and less than 10% at 72–84 km. The relative differences of the H${}_{2}$O profile mostly stayed within the acceptable range of MLS differences above 16 km for 65${}^{\circ }$–70${}^{\circ }$ S. However, the relative differences were a little larger below 16 km. The relative differences of the H${}_{2}$O profiles were the same as those shown in Figure 9, which indicates that the relative differences below 16 km were mainly caused by the large uncertainties of measurement at lower latitudes.

(3) 70${}^{\circ }$–75${}^{\circ }$ S

**Figure 14:**
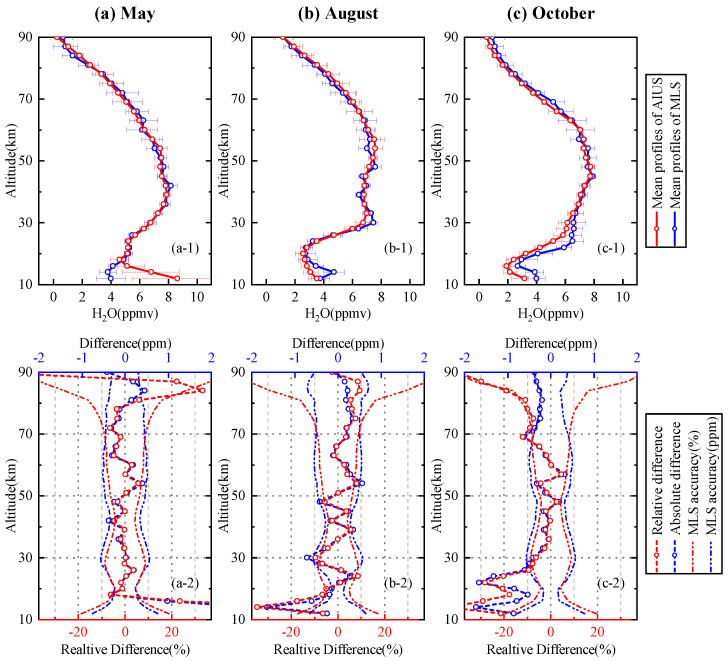
Same as Figure 12 except for 70${}^{\circ }$–75${}^{\circ }$ S in May, August, and October.

Figure 14 presents comparison of the H${}_{2}$O profiles for 70${}^{\circ }$–75${}^{\circ }$ S in May, August, and October. Figure 14a-1,b-1 illustrate that the mean profile of AIUS had a good agreement with that of MLS in both May and August. The AIUS profile had an obviously negative deviation from MLS below 30 km in October, shown in Figure 14c-1. Figure 14a-2,b-2,c-2 show that the relative differences were mostly within ±6.5% at 18–81 km in May, ±9.6% at 18–90 km in August, and ±12% at 26–81 km in October. The accuracy of H${}_{2}$O retrievals at lower altitudes for 70${}^{\circ }$–75${}^{\circ }$ S is not as good as that of 65${}^{\circ }$–70${}^{\circ }$ S. The reasons for this need to be further explored. In the future, more data will be used for analysis.

(4) 75${}^{\circ }$–80${}^{\circ }$ S

**Figure 15:**
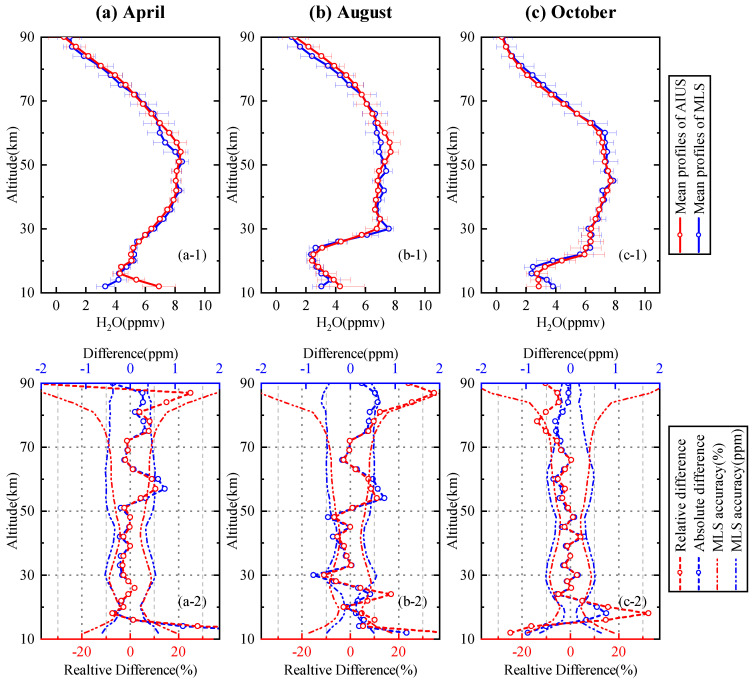
Same as Figure 12 except for 75${}^{\circ }$–80${}^{\circ }$ S in April, August, and October.

Figure 15 presents a comparison of the H${}_{2}$O profiles for 75${}^{\circ }$–80${}^{\circ }$ S in April, August, and October. Figure 15a-1,b-1,c-1 illustrate that the mean profile of AIUS agreed well with that of MLS in all three months, which indicated that that the vertical distribution of the H${}_{2}$O profile was captured well by AIUS measurements. The standard deviations of the AIUS H${}_{2}$O profiles in August were slightly larger than the other two months. Figure 15a-2,b-2,c-2 show that the differences were within ±4.5% at 16–54 km and −2% to 11% at 54–81 km in April, ±10% at 14–78 km in August, ±6% at 22–72 km, and ±15% at 14–20 km in October. In general, the differences mostly stayed within the acceptable range of MLS differences at 16–90 km for 75${}^{\circ }$–80${}^{\circ }$ S.

## 5. Discussion

The assessment of AIUS spectra was illustrated by comparing the measurements with simulations. The differences between AIUS measurements and simulations at lower altitudes are larger than those at higher altitudes. This suggests that the differences are related to the increased pressure and pressure broadening at lower altitudes. The larger differences can be seen in the 980–1095 cm${}^{-1}$, 2250–2400 cm${}^{-1}$, 2800–3200 cm${}^{-1}$, and 3500–3750 cm${}^{-1}$. The absorptions are dominated by O${}_{3}$, CO${}_{2}$, CH${}_{4}$, and CO${}_{2}$, respectively, in these spectral bands. This might be mainly caused by the uncertainties of measurement during observation and the uncertainties of the *a priori* profiles. In addition, the signal-to-noise ratio is less than 200 in these spectral bands, which may also lead to large transmittance differences.

The H${}_{2}$O profiles were retrieved from AIUS measurements and then compared with MLS Level 2 products. The H${}_{2}$O retrieval experiments were carried out at 12–90 km as the quality of the measurements beyond the altitude range was unacceptable. The comparisons show that the mean AIUS H${}_{2}$O profiles are similar to the mean MLS profiles, while larger relative differences of H${}_{2}$O profiles can be found below 16 km. One reason for the larger relative difference may be the large uncertainties of AIUS measurement in lower altitudes. Another reason might come from the uncertainties of the tangent altitudes below 20 km. The comparison also revealed that the differences of H${}_{2}$O profiles were larger in the mesosphere. This could be caused by the larger uncertainty of the official MLS products above 60 km.

## 6. Conclusions

In this study, the spectral quality assessment of AIUS measurements and the validation of H${}_{2}$O retrievals were demonstrated. The transmittance of AIUS measurements were compared with that of simulated spectra and ACE-FTS measurements. The mean and standard deviations of transmittance differences between AIUS measurements and simulated spectra were then calculated. It illustrated that AIUS measurements were mostly consistent with simulations and ACE-FTS measurements. Therefore, AIUS measurements can be used for O${}_{3}$, H${}_{2}$O, temperature, CO, CH${}_{4}$, and HCl detection. The validation of H${}_{2}$O profiles was performed using 153 pairs of AIUS and equivalent MLS measurements in four latitude zones of 60${}^{\circ }$–65${}^{\circ }$ S, 65${}^{\circ }$–70${}^{\circ }$ S, 70${}^{\circ }$–75${}^{\circ }$ S, and 75${}^{\circ }$–80${}^{\circ }$ S. The experiment revealed that the precision of H${}_{2}$O retrievals is comparable to that of MLS. The relative differences of monthly mean profiles between AIUS and the equivalent MLS are mostly within ±10% at 16–75 km and about 10–15% at 16–20 km in 60${}^{\circ }$–80${}^{\circ }$ S. It revealed that the relative differences mostly stayed within the acceptable range of the MLS differences at 16–90 km. Thus, the H${}_{2}$O profiles retrieved from AIUS are credible and can be used for scientific research.

AIUS is the first hyperspectral occultation spectrometer in China. The noise level is still slightly higher in 980–1095 cm${}^{-1}$ and 3500–3750 cm${}^{-1}$. Presently, the relative differences of the H${}_{2}$O retrievals were large below 16 km. In the future, further studies are required to improve the method of H${}_{2}$O retrieval in the lower altitudes to reduce the impact of uncertainties of measurement on H${}_{2}$O retrievals. In addition, more measurements are required to validate the accuracy of the H${}_{2}$O profile and other trace gases profiles. Further validation will be performed by comparing the mean profiles with more correlative data from space-borne, ground-based, and balloon platforms. 

## Figures and Tables

**Table 1:** Information about the AIUS measurements.

Latitude Zone	Month	Number
60${}^{\circ }$–65${}^{\circ }$ S	January	7
	December	17
65${}^{\circ }$–70${}^{\circ }$ S	June	13
	July	14
	November	16
70${}^{\circ }$–75${}^{\circ }$ S	May	14
	August	15
	October	12
75${}^{\circ }$–80${}^{\circ }$ S	April	16
	August	14
	October	15

**Figure A1:**
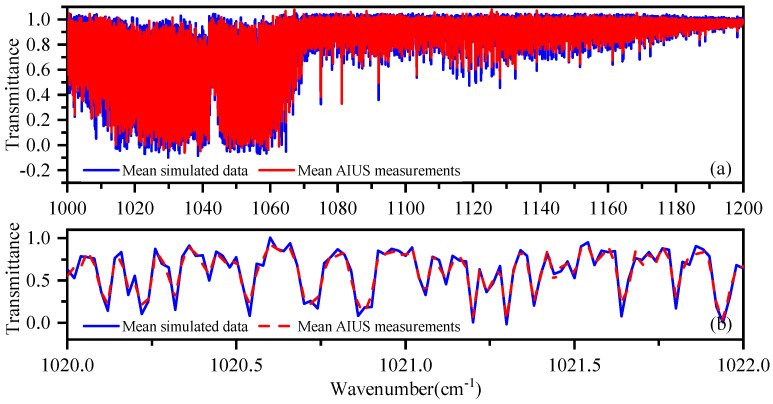
Mean transmittance of AIUS measurements and simulations in 1000–1200 cm${}^{-1}$ at 40 km.

**Figure A2:**
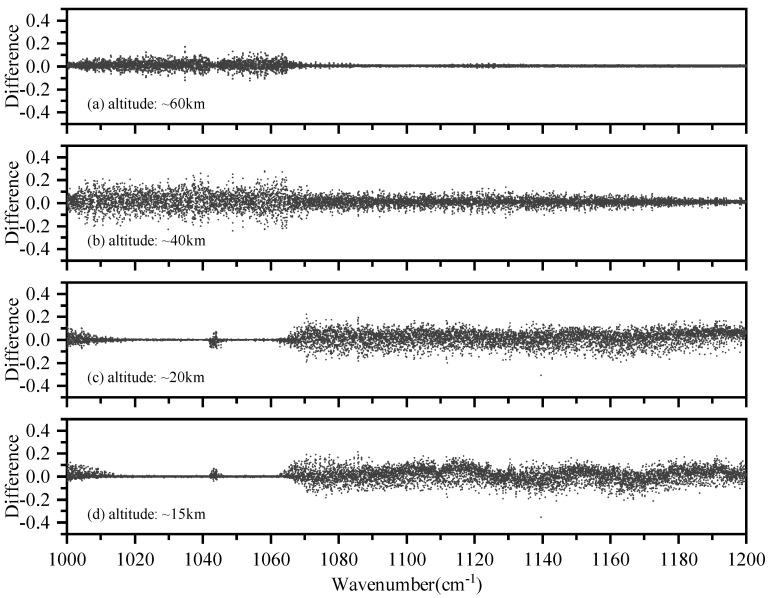
The transmittance differences between AIUS measurements and simulations in 1000–1200 cm${}^{-1}$.

**Figure A3:**
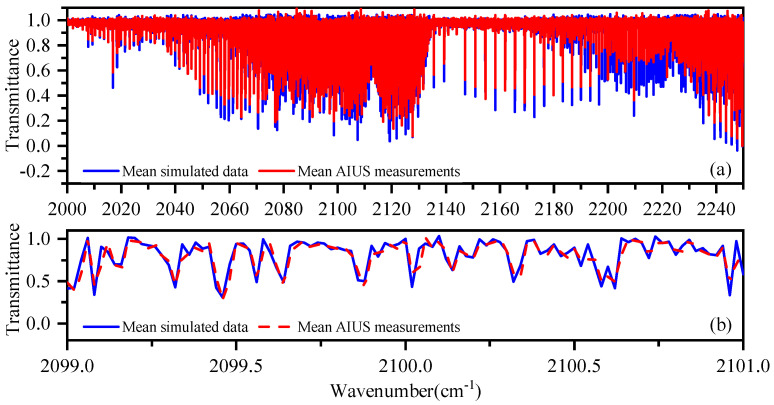
Mean transmittance of AIUS measurements and simulations in 2000–2250 cm${}^{-1}$ at 40 km.

**Figure A4:**
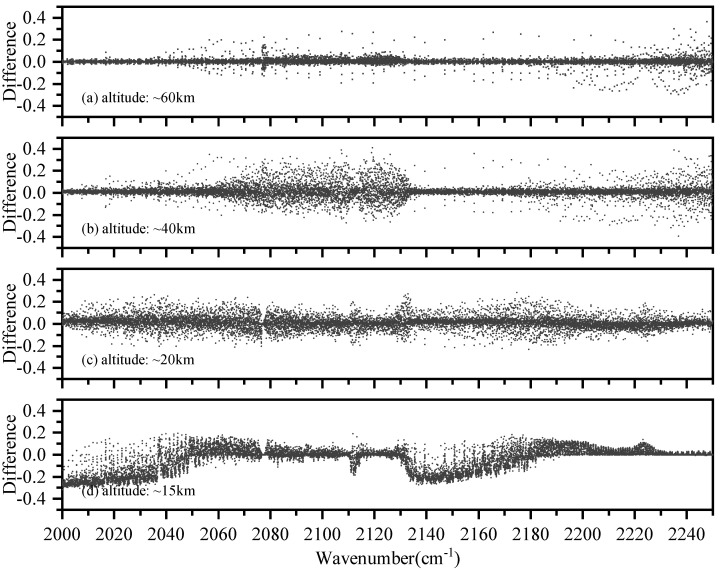
The transmittance differences between AIUS measurements and simulations in 2000–2250 cm${}^{-1}$.

**Figure A5:**
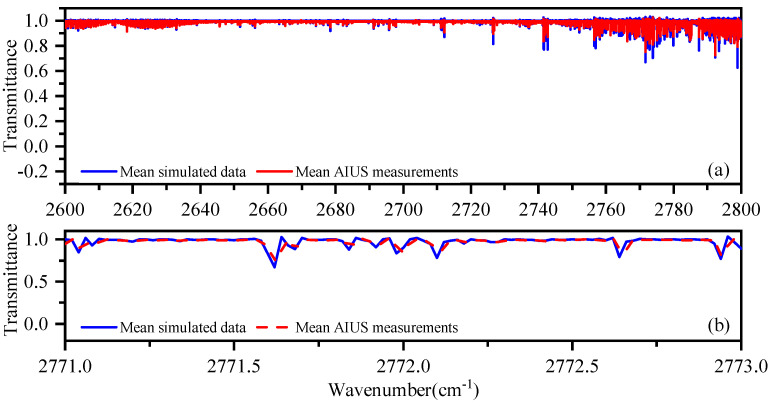
Mean transmittance of AIUS measurements and simulations in 2600–2800 cm${}^{-1}$ at 40 km.

**Figure A6:**
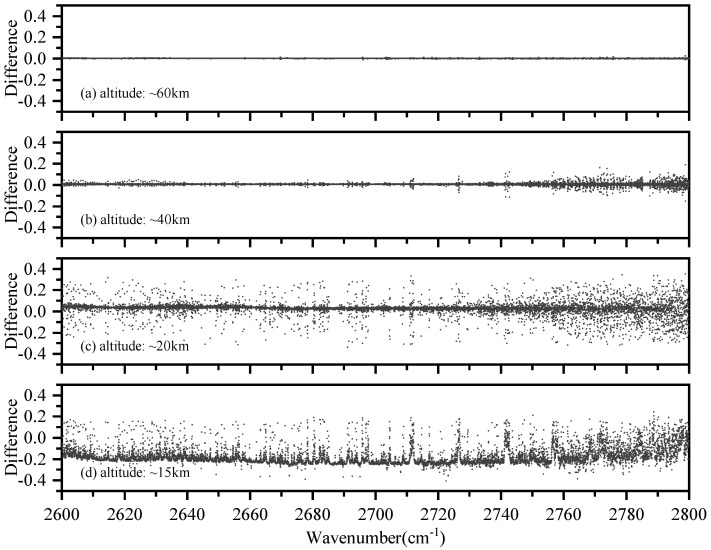
The transmittance differences between AIUS measurements and simulations in 2600–2800 cm${}^{-1}$.

**Figure A7:**
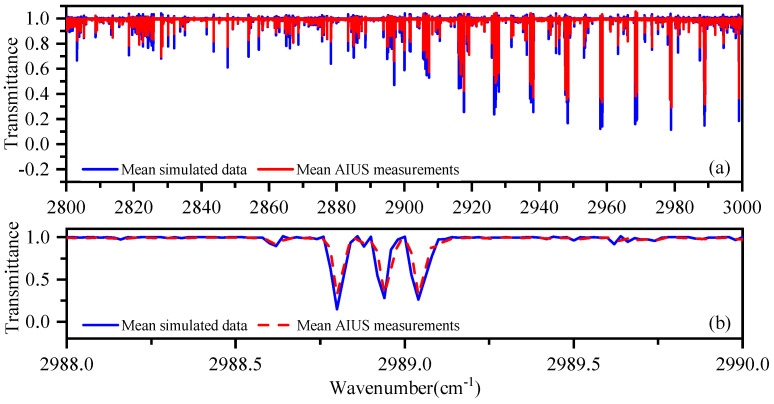
Mean transmittance of AIUS measurements and simulations in 2800–3000 cm${}^{-1}$ at 40 km.

**Figure A8:**
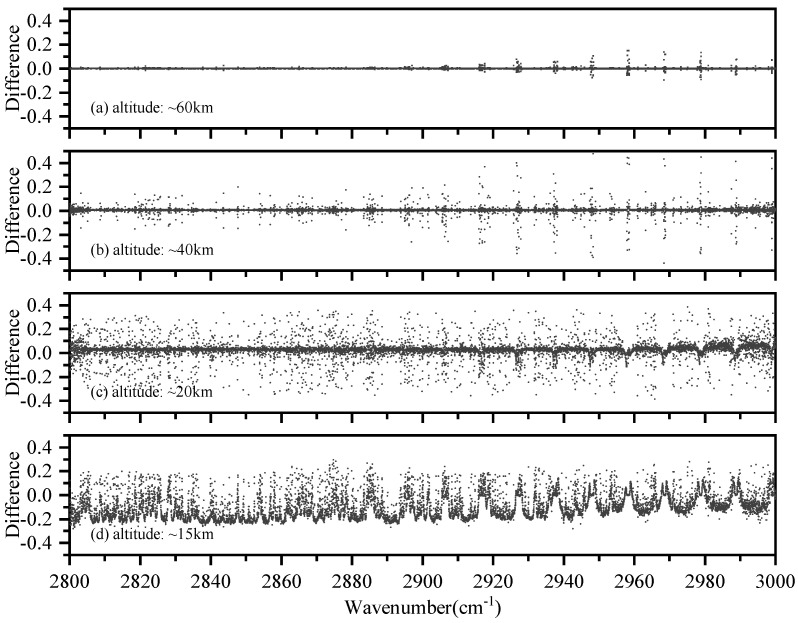
The transmittance differences between AIUS measurements and simulations in 2800–3000 cm${}^{-1}$.

**Figure A9:**
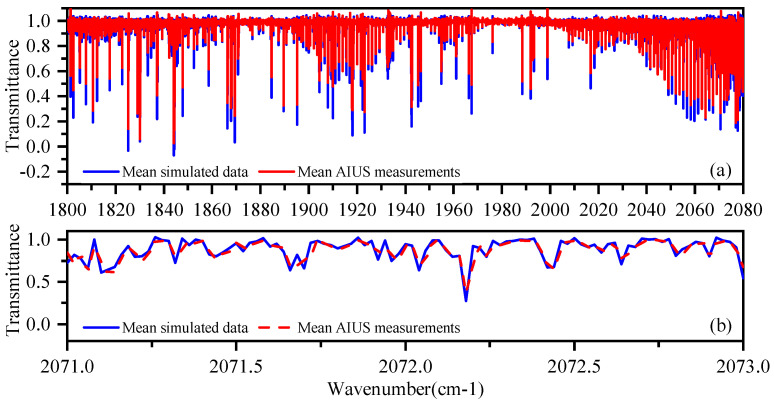
Mean transmittance of AIUS measurements and simulations in 1800–2080 cm${}^{-1}$ at 40 km.

**Figure A10:**
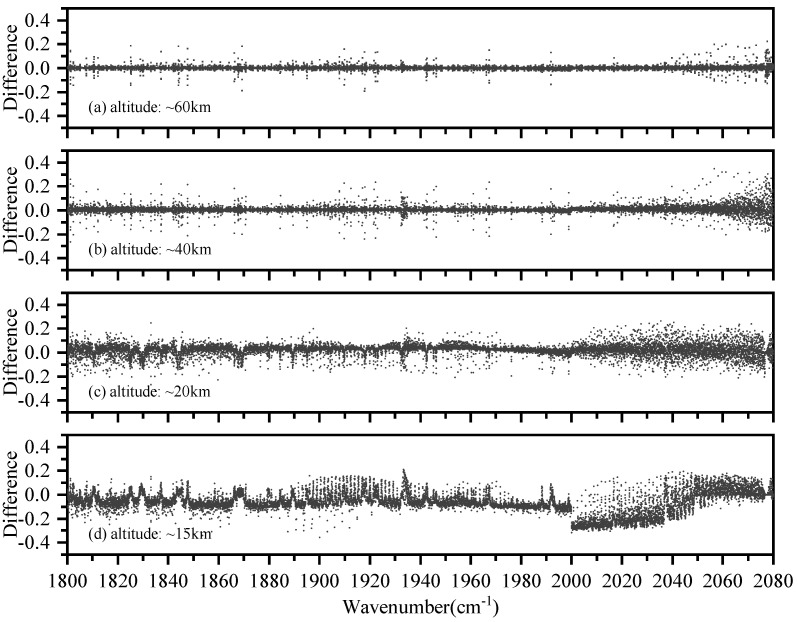
The transmittance differences between AIUS measurements and simulations in 1800–2080 cm${}^{-1}$.

## Data Availability

Data sharing not applicable.

## Appendix A

Comparisons were also made between AIUS measurements and simulations in the spectral windows of O${}_{3}$, CO, CH${}_{4}$, HCl, and temperature.

(1) 1000–1200 cm${}^{-1}$ for O${}_{3}$

Figure A1 presents the mean transmittance of AIUS measurements and simulations in 1000–1200 cm${}^{-1}$ at 40 km, where absorptions are dominated by O${}_{3}$, H${}_{2}$O, CH${}_{4}$, N${}_{2}$O, and HNO${}_{3}$. O${}_{3}$ can be detected and retrieved by this spectral window. Figure A1a illustrates that the AIUS measurements had a good agreement with simulations. Figure A1b shows the spectra in a zoom-in window centered at 1021 cm${}^{-1}$. The absorption peaks of measurements agreed well with those of simulations. Therefore, AIUS can provide reliable data for O${}_{3}$ detection and dynamic monitoring.

Figure A2 shows the transmittance differences between AIUS measurements and simulations in 1000–1200 cm${}^{-1}$ at 15 km, 20 km, 40 km, and 60 km, respectively. Figure A2a indicates that the transmittance differences were mostly within ±0.1 in 1000–1065cm${}^{-1}$ and ±0.05 in 1065–1200 cm${}^{-1}$ at 60 km. Figure A2b shows that the transmittance differences were within ±0.2 in 1000–1065 cm${}^{-1}$ and ±0.1 in 1065–1200 cm${}^{-1}$ at 40 km. Figure A2c,d show that the transmittance differences were within ±0.1 in 1000–1060 cm${}^{-1}$ and ±0.2 in 1060–1200 cm${}^{-1}$ at 40 km and 20 km. In 1000–1060 cm${}^{-1}$, the larger differences were found at 40 km where the absorption was strong. In 1080–1200 cm${}^{-1}$, the transmittance differences decreased with altitude increasing.

(2) 2000–2250 cm${}^{-1}$ for CO

Figure A3 shows the comparison between AIUS measurement and simulation in 2000–2250 cm${}^{-1}$ at 40 km. CO, N${}_{2}$O, and H${}_{2}$O are the main absorbers in this spectral window. It can be used for CO detection. Figure A3a shows the spectra of measurement and simulation, which were similar to each other. Figure A3b shows the spectra in a zoom-in window centered at 2100 cm${}^{-1}$. The absorption peaks of measurements agreed well with those of the simulations.

Figure A4 presents the transmittance differences variation with altitudes (15 km, 20 km, 40 km, and 60 km) in 2000–2250 cm${}^{-1}$. Figure A4a illustrates that the transmittance differences were within ±0.1 in 2000–2040 cm${}^{-1}$ and ±0.3 in 2040–2250 cm${}^{-1}$ at 60 km. Figure A4b illustrates that the transmittance differences were within ±0.2 in 2000–2050 cm${}^{-1}$ and −0.3 to 0.4 in 2050–2250 cm${}^{-1}$ at 40 km. Figure A4c illustrates that the transmittance differences were within ±0.25 at 20 km, with 90% of the differences within ±0.1. Figure A4d illustrates that the transmittance differences were in the range of −0.3 to 0.2 in 2000–2200 cm${}^{-1}$ and −0.01 to 0.1 in 2200–2250 cm${}^{-1}$ at 15 km. Generally, in 2000–2050 cm${}^{-1}$ and 2135–2180 cm${}^{-1}$, the transmittance differences at lower altitudes were larger than at higher altitudes. In 2200–2250 cm${}^{-1}$, the transmittance differences were larger at higher altitudes.

(3) 2600–2800 cm${}^{-1}$ for CH${}_{4}$

Figure A5 shows the comparison between AIUS measurement and simulation in 2600–2800 cm${}^{-1}$ at 40 km. CH${}_{4}$ and N${}_{2}$O are the main absorbers in this spectral window. It can be used for CH${}_{4}$ detection. Figure A5a shows that the measurements had a good agreement with simulations in 2600–2800 cm${}^{-1}$. Figure A5b shows the spectra in a zoom-in window centered at 2772 cm${}^{-1}$. The absorption peaks of measurements agreed well with those of the simulations.

Figure A6 presents the transmittance differences variation with altitudes (15 km, 20 km, 40 km, and 60 km) in 2600–2800 cm${}^{-1}$. Figure A6a illustrates that the transmittance differences can be ignored at 60 km. Figure A6b illustrates that the transmittance differences were mostly within ±0.05 in 2600–2750 cm${}^{-1}$ and ±0.1 in 2750–2800 cm${}^{-1}$ at 40 km. Figure A6c illustrates that the transmittance differences were within ±0.3 at 20 km, with 79% of the differences within ±0.05. Figure A6d illustrates that the transmittance differences were mostly in the range of −0.2 to 0.2 at 15 km. In general, the transmittance differences were larger at lower altitudes in 2600–2800 cm${}^{-1}$.

(4) 2800–3000 cm${}^{-1}$ for HCl

Figure A7 shows the comparison between AIUS measurement and simulation in 2800–3000 cm${}^{-1}$ at 40 km. The absorptions are dominated by HCl, N${}_{2}$O, CH${}_{4}$, and NO${}_{2}$. It can be used for HCl detection. Figure A7a shows the measurements agreed well with simulations. Figure A7b shows the spectra in a zoom-in window centered at 2989 cm${}^{-1}$. The absorption peaks of measurements were consistent with those of the simulations.

Figure A8 presents the transmittance differences variation with altitudes (15 km, 20 km, 40 km, and 60 km) in 2800–3000 cm${}^{-1}$. Figure A8a illustrates that the transmittance differences were in the range of −0.1 to 0.15 at 60 km. Figure A8b illustrates that the transmittance differences were within ±0.4 at 40 km, with 96% of the differences within ±0.05. Figure A8c shows that the transmittance differences were within ±0.4 at 20 km, with 88% of the differences within ±0.1. Figure A8d shows that the transmittance differences were within ±0.3 at 15 km, with 60% of the differences within ±0.15. In general, the transmittance differences were larger at lower altitudes in 2800–3000 cm${}^{-1}$.

(5) 1800–2080 cm${}^{-1}$ for temperature

Figure A9 presents the mean transmittance of AIUS measurements and simulations in 1800–2080 cm${}^{-1}$ at 40 km, where absorptions are dominated by CO${}_{2}$, H${}_{2}$O, H${}_{2}$O, NO, etc. This can be used for temperature detection and retrieval, as the CO${}_{2}$ concentration is relatively stable. Figure A9a shows the measurements agreed well with simulations in 1800–2080 cm${}^{-1}$. Figure A9b shows the spectra in a zoom-in window centered at 2072 cm${}^{-1}$. The absorption peaks were mostly consistent with each other, with a slight deviation in 2071.7 cm${}^{-1}$, 2072.04 cm${}^{-1}$, 2072.64 cm${}^{-1}$, etc.

Figure A10 presents the transmittance differences between the measurements and simulations in 1800–2080 cm${}^{-1}$ at 15 km, 20 km, 40 km, and 60 km. Figure A10a shows that the transmittance differences were mostly within ±0.2 at 60 km. Figure A10b shows that the transmittance differences were mostly within ±0.3 at 40 km, with 94% of the differences were within ±0.05. Figure A10c illustrates that the transmittance differences were within ±0.3 at 20 km, with 94% of the differences within ±0.1. Figure A10d illustrates that the transmittance differences were within −0.4 to 0.2 at 15 km, with 66% of the difference within ±0.15. There was a sudden difference reduction at 2000 cm${}^{-1}$ in Figure A10d. The reason was that the transmittance around 2000 cm${}^{-1}$ was obtained by different detectors (MCT and InSb). This indicates that the performance of the two detectors was different near 2000 cm${}^{-1}$ at 15 km.
